# Analysis of a Driving Simulator’s Steering System for the Evaluation of Autonomous Vehicle Driving

**DOI:** 10.3390/s25206471

**Published:** 2025-10-20

**Authors:** Juan F. Dols, Samuel Boix, Jaime Molina, Sara Moll, Francisco J. Camacho, Griselda López

**Affiliations:** 1 Design and Manufacturing Institute (IDF), Universitat Politècnica de València, 46022 Valencia, Spain; jaimopar@idf.upv.es; 2 Highway Engineering Research Group (HERG), Universitat Politècnica de València, 46022 Valencia, Spain; sboitor@upv.edu.es (S.B.); samolmon@upvnet.upv.es (S.M.); fracator@tra.upv.es (F.J.C.); grilomal@tra.upv.es (G.L.)

**Keywords:** driving simulator, autonomous driving, steering system, calibration evaluation, automated simulation, virtual scenario development

## Abstract

**Highlights:**

**What are the main findings?**

- The EVACH driving simulator, equipped with custom hardware and data acquisition systems, achieved precise calibration of the braking and steering controls.
- Comparative analysis between naturalistic tests and virtual simulations showed that the simulator reproduces autonomous driving speeds with fidelity and stability.

**What is the implication of the main finding?**

- The EVACH simulator provides a robust and reliable platform for investigating driver behavior and human–machine interaction in SAE 2 and SAE 3 scenarios.
- Its validated calibration and reliable virtual environment enable safe, cost-effective, and versatile experimentation for future autonomous driving research projects.

**Abstract:**

The integration of autonomous vehicles (AVs) into road transport requires robust experimental tools to analyze the human–machine interaction, particularly under conditions of system disengagement. This study presents the primary controls calibration and virtual scenario validation of the EVACH autonomous driving simulator, designed to reproduce the SAE Level 2 and Level 3 driving modes in rural road scenarios. The simulator was customized through hardware and software developments including a dedicated data acquisition system to ensure the accurate detection of braking, steering, and other critical control inputs. Calibration tests demonstrated high fidelity, with minor errors in brake and steering control measurements, consistent with values observed in production vehicles. To validate the virtual driving rural environment, comparative experiments were conducted between naturalistic road tests and simulator-based autonomous driving, where five volunteers participated in the preliminary pilot test. Results showed that average speeds in the simulation closely matched those recorded on real roads, with differences of less than 1 km/h with minimum standard deviation and confidence values. These findings confirm that the EVACH simulator provides a stable and faithful reproduction of autonomous driving conditions. The experimental platform offers valuable support for current and future research on the safe deployment of automated vehicles.

## 1. Introduction

The impact of human factors on traffic accidents is well-known. The deployment of autonomous vehicles (AVs) is expected to lead to improved road safety, associated with a reduction in accidents due to driver inattention or distractions [1]. In 2016, the Society of Automotive Engineers (SAE) published a classification that distinguished between six levels of autonomous driving [2]. This classification ranges from level 0, which refers to completely manual driving with no automation in the vehicle, to level 5, which represents fully autonomous driving. Intermediate levels are defined based on the person performing the driving task (human or machine) and the specific operating conditions of the system.

The most advanced systems currently available on the market correspond to automation levels SAE 2 and SAE 3 [3]. At level SAE 2, the vehicle is capable of autonomously controlling lateral and longitudinal movements simultaneously [4]. Longitudinal control allows the lane keeping assist (LKA) autonomous driving system to control speed, detect the preceding vehicle, keep a safe distance, and apply the accelerator or brake, as needed. These are the functions of the adaptive cruise control (ACC) system. Regarding lateral control, the autonomous driving system detects the vehicle’s position relative to the lane edges. There are basically two behaviors: LKA and lane centering assist (LCA). In both cases, the system can control the vehicle’s direction, but while the LCA system attempts to keep the vehicle centered in the lane, the LKA system only performs corrective steering actions if the driver tends to stray. The operation of these functionalities is fundamentally based on reading road markings, which are used by the system to guide and maintain the vehicle in the lane [5]. At the SAE 2 automation level, the driver is required to remain in a state of constant alert, as the automated driving system may disengage suddenly, immediately transferring full control of the vehicle to the human driver.

At the SAE 3 automation level, the autonomous driving system maintains functional similarities with the SAE 2 level—it simultaneously controls the longitudinal and lateral position of the vehicle, but the system can anticipate to potential disengaging situations. This is communicated to the human driver by issuing a take-over request (TOR) as soon as the disengagement is foreseen so that they can regain control of the driving task. In this context, the driver can perform non-driving-related tasks (NDRTs) during automated operation. However, this condition can compromise safety, as the transition of control—from the autonomous system to the driver—can be compromised by the limited interaction between the driver and the vehicle, especially if the driver is not fully prepared to regain control in a timely and safe manner [6,7,8,9].

Autonomous driving greatly influences the drivers’ workload demand [10]. Workload can be defined as the level of difficulty experienced by a driver in performing their driving task [11]. The SAE 2 and SAE 3 levels aim to reduce the workload demand. However, some studies suggest that system errors and frequent system disengagements may increase the workload because the driver needs to continuously monitor its operation [12]. Furthermore, low workload demand can be a safety issue if a disengagement occurs when the driver is distracted [13]. Therefore, distractions and attention reduction may negatively affect the AVs’ takeover performance when they are asked to take control of the vehicle [14,15].

Until full automation is achieved, it is essential to address two key issues: (i) the influence of the frequency of automated driving system disengagements at the SAE 2 and SAE 3 levels on driver behavior and cognitive workload, and (ii) the conditions required to ensure a safe transition of vehicle control when the driver is engaged in non-driving-related tasks (NDRT), particularly at the SAE 3 level.

Recent studies have examined the phenomenon of take-over requests, with particular emphasis on driver reactions [16,17]. However, most of them have focused on isolated disengagement situations. It is therefore important to investigate engagement and disengagement processes over longer road segments, where drivers can alternate between manual and automated driving.

Automated driving system disengagements typically occur when the vehicle leaves the operational design domain (ODD) for which it was designed, or because of unexpected failures in the system itself. These disengagements may be triggered by multiple factors including infrastructure characteristics (such as horizontal and vertical alignments, cross section, pavement condition, and road markings), limitations of the vehicle itself (technological capabilities, operating errors, etc.) as well as external variables such as weather or visibility [13,18,19,20].

At the same time, it is essential to investigate the driver’s response to these disengagements. This requires the development of specific studies aimed at understanding, predicting, and managing the interactions between the driver and the vehicle, with the goal of ensuring a transition from autonomous driving mode to manual mode as safe, smooth, and efficient as possible.

Driving simulators are a key tool for evaluating driver behavior, as they allow for greater control over the test scenario while avoiding safety issues. Thanks to their advantages in terms of safety, cost-effectiveness, and the ability to control variables in experiments, driving simulators have been widely used in the research of human factors in autonomous driving [21,22,23,24,25,26,27,28]. Cheng et al. [29] further demonstrated their utility in risky traffic environments, supporting the application of this approach in the present study, where AV disengagements are considered high-risk events.

Although moving-base simulators are generally preferred, fixed-base simulators are also considered valid [30]. Simulator validity depends on factors such as horizontal field of view, motion system, and in-car versus out-of-car configuration. The EVACH simulator is a fixed-base, in-car simulator with a 180° horizontal field of view. According to [30], it shows relative validity for measures such as the standard deviation of the headway and standard deviation of the lane position, thereby confirming its research utility.

In addition, even though dynamic simulators provide more motion feedback and a higher level of immersion, previous studies have also demonstrated that fixed-base simulators can still yield valid and reliable results for the analysis of driver behavior and human–machine interaction. Validation studies have shown consistent findings for visual attention, reaction times, distraction effects, speed profiles, and road geometric influences [31,32,33,34].

Furthermore, driving simulator testing allows for the use of physiological sensors in a controlled environment and can be performed at relatively low costs. Deng et al. [35] analyzed physiological responses to different types of driver behavior (brain signals, skin conductance level—SCL, and heart rate—HR) during the takeover phases of autonomous driving, finding remarkable insights into the patterns of physiological data changes during takeover periods. Tomasevic et al. [36] validated the use of driving simulators as a tool to analyze the human factors associated with autonomous vehicles, although their study did not consider control changes between the driver and the automated system.

More recently, Sekadakis et al. [37] used a driving simulator to characterize different types of driving behaviors during and after the takeover phases in SAE levels 2 and 3, concluding that driver response was primarily influenced by individual driver behavior rather than the level of automation itself.

Nevertheless, most of the driving simulator studies above-mentioned have only experienced very specific episodes of disengagement from the autonomous driving system. These attempt to reproduce experiments in which the driver assumes control of the vehicle occasionally in response to sudden events in the virtual environment [38,39]. However, few studies have analyzed driver behavior focused on continuously trying to hand over control from and to the autonomous driving system in the virtual simulator environment.

The high ecological validity of naturalistic data for assessing driver behavior has been demonstrated in several studies [40,41,42]. The EVACH simulator developed in this study was designed and calibrated using naturalistic data collected by AV trips on a Spanish rural road. It reproduces both manual and automated driving modes along extended roadways segments, with special attention to mode transitions. A scenario presenting autonomous- and manual-ready zones was created to provide participants with driving experiences closely resembling real conditions.

To this end, the experimental tool used during the simulation of autonomous driving must be designed to faithfully and reliably reproduce the behavior of an autonomous vehicle during the takeover and transfer of driving control from the simulator (system) to the driver. In these cases, the driving simulator’s main controls (acceleration and braking pedals, steering system) must be designed, configured, and programmed to automatically adjust to manual or autonomous driving modes, depending on the needs of the experiment to be reproduced.

This is precisely one of the issues barely discussed in the scientific literature, where the design and reliability conditions of driving simulators used in autonomous driving studies are not fully described. This research is part of the EVACH research project, funded by the Research Spanish Agency (RSA) and European FEDER funds. It dedicates a significant part of its activities to the development of a driving simulator designed to reproduce both current and future levels of vehicle automation. In addition to the simulator sensors, an existing scenario has been updated to include its autonomous driving readiness along its path. This scenario is already a digital twin of a real road. With this new information, the driving simulator is expected to recreate the autonomous and manual behavior at the same zones that an actual vehicle does along the real road. The main objective of this driving simulator is to evaluate human behavior and analyze human–machine interaction in situations characterized by different frequencies of disengagements. Performing these evaluations is essential to ensure the overall safety of the integrated system.

Therefore, the objective of this article is to describe the construction elements, sensors, and instrumentation used to adapt and control a driving simulator for conducting experiments that allow for versatile and customizable reproduction of the SAE 2 and SAE 3 manual and autonomous driving modes in adaptable simulation environments. This approach will be applied in this work to calibrate the driving simulator controls and validate the virtual scenario by simulating a rural road section of sufficient length (30 km) to define multiple subsections in which the autonomous driving system remains operational, interspersed with other subsections in which it disengages. In this way, the driver is forced to repeatedly hand over and take over, allowing for a more comprehensive study of the dynamics of human–machine interaction in intermittent autonomous driving situations.

## 2. Materials and Methods

**Figure 1:**
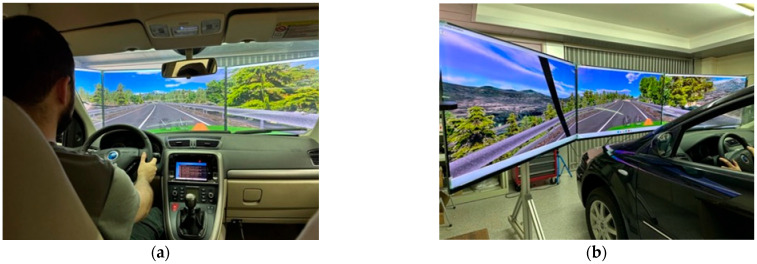
(**a**) Interior view of the EVACH driving simulator. (**b**) Exterior view of the EVACH driving simulator.

The driving simulator used for the EVACH project was initially developed to assess the driving ability of people with or without motor disabilities and has been used in numerous research projects focused on driver assessment [43] as well as in the passive safety assessment of adapted vehicles [44,45]. In the context of the development of the EVACH project and to recreate an autonomous driving model, it has become necessary to specifically redesign and adapt both the simulation software and the data acquisition hardware. The simulation platform uses the complete body of a 2007 Fiat Croma, equipped with a computer with an Asus TUF Z790-PLUS WIFI motherboard, Intel Core i9-14900K processor, Kingston Fury Beast RGB 16 GB RAM, Zotac GeForce RTX 4070 Ti graphics card, and Corsair MP600 1 TB PCIe 4.0 storage. It also has a signal acquisition card (Nucleo-H743ZI from STMicroelectronics), sensors for measuring the position of the accelerator, brake, and clutch pedals (Allegro UGN3503UA), an electric motor for steering control (MAXON RE-40) and its controller (MAXON ADS 50/10 201583), a sensor for measuring torque (HBM T20W) and the steering position, a load cell for acquiring the force applied during braking (INTERFACE WMC-500), and a power supply for the different electronic systems as well as a graphic display system consisting of three 55” 4K Samsung screens (UW55AU7025) and a 7” video monitor (800 × 480) installed inside the cabin. Figure 1 shows an interior and exterior view of the EVACH autonomous driving simulator.

For the EVACH project, a steering control system was implemented during virtual simulation that emulates the behavior of an autonomous vehicle, allowing the steering system to reproduce the path the vehicle always follows in real traffic. Autonomous driving mode requires the control of several parameters related to the primary controls such as:

- Steering system equipment: Torque applied to the steering system, steering wheel angle and steering wheel centering signal;
- Pedal position: Position of the brake and accelerator pedals;
- Pedal forces: Pedal actuation and applied force.

**Figure 2:**
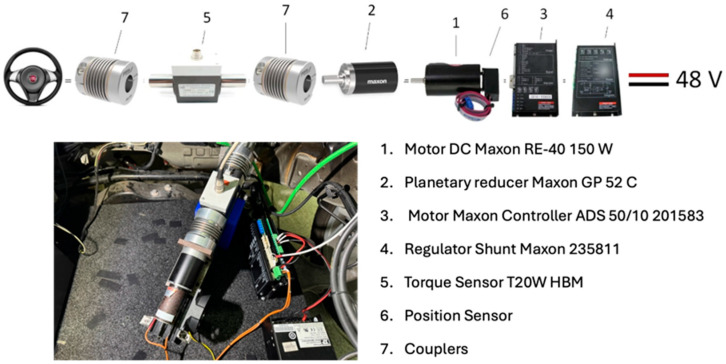
Components of the steering and control system in the simulator.

Figure 2 identifies the different elements of the steering control system implemented in the simulator. The Maxon RE-40 motor (1) is responsible for generating the movement required by the system to recreate autonomous driving levels. It has a power of 150 W at 48 V nominal and 40 mm in diameter. To achieve greater torque, the system includes a planetary reducer (2) from the manufacturer Maxon, model GP 52 with a reduction ratio of 1:27. The MAXON 201583 controller (3), a servo amplifier device suitable for controlling motors from 80 W to 500 W, offers the possibility of controlling the speed, torque, and protection against overcurrent, excess temperature, and short circuits in the motor winding. The MAXON 235811 shunt regulator (4) is designed to limit the amplifier supply voltage and help maintain a constant voltage. In addition, the engine control system incorporates an encoder (6) that measures the speed and angle of rotation of the steering wheel. The HBM T20W torque sensor (5) installed can transmit up to 50 Nm of nominal torque. The flexible helical spring couplers (7) allow for the connection between shafts that will transmit torque, and in this case, ensure the correct transmission of movement between the components of the steering kinematic chain (cardan joint), the torque sensor, and the electric motor.

To determine the steering wheel angle, a steering centering sensor was installed. This consisted of a custom-designed unipolar Hall effect sensor from the manufacturer Melexis US5881. This sensor detects centering by installing a magnet attached to the moving element. The brake and accelerator pedal positions require knowledge of their relative position to determine the vehicle’s dynamic behavior (acceleration/braking). In this case, ad hoc sensors based on the radiometric Hall effect were designed for this simulator. UGN3503UA sensors from the manufacturer Allegro were used to position the brake and clutch pedals, capable of providing output voltages based on the angle of rotation of each pedal. A WMC-500 load cell manufactured by INTERFACE, with a capacity of 2224.1 N (500 lbf), was used to measure the degree of braking force applied.

The chosen data acquisition and processing system is based on the ARM^®^ Cortex^®^ M7 microcontroller family. Specifically, STM32H743ZIT6U from the manufacturer STMicroelectronics was selected. This microcontroller integrates analog-to-digital converters with up to 12 channels per unit. It also has four USART (universal synchronous/asynchronous receiver/transmitter) serial communication ports and four UART (universal asynchronous receiver/transmitter) ports. Additionally, it has a USB-enabled communication peripheral that is capable of emulating a CDC (communications device class) port, enabling the fast and secure transmission of information between the simulator and the microcontroller.

The logic core of the system was implemented on an STM32 board, where a control scheme based on a PID regulator has been programmed in C language. This algorithm compares the recorded position and torque in real-time with the target values from the simulator, dynamically adjusting the signal sent to the motor. The board selected for the data acquisition system was the STMicroelectronics NUCLEO-H743ZI model, which includes a USB port for programming and debugging, an additional USB port for connecting to the simulator, and a 10/100 Mb LAN port in case greater bandwidth and stability are required in the communication between the simulator and the acquisition system.

Thus, the combination of the T20WN sensor, the centering sensor, the motor with its controller, and the digital control loop implemented in C allows the steering commands generated by the simulator to be reliably executed. The system ensures that the steering wheel follows the trajectory established in each scenario, guaranteeing a smooth transition between manual and autonomous driving modes within the experimental platform.

Therefore, to reliably reproduce the SAE 2 and SAE 3 driving modes in the simulator, the operating state of the steering control system was classified into two driving modes: (i) manual (the simulator receives input from the driver, who handles the primary controls and directly controls the vehicle’s trajectory) and (ii) autonomous (the vehicle operates without driver intervention, with the software sends the parameters of speed and position relative to the central axis of the road).

In autonomous operating mode, the control software acts directly on the simulator hardware and allows the vehicle to operate autonomously in the virtual environment without the driver having to perform any actions on the pedals and steering wheel. Changes between manual and autonomous operating modes occur either when the driver applies certain actions to the controls, or when the control system detects that the vehicle has entered certain areas of the virtual environment where autonomous mode is deactivated.

The simulator’s steering control system was designed so that during manual driving mode, the force the driver must exert on the periphery of the steering wheel allows for an action on the steering system identical to that found in current market automobiles [46,47]. In autonomous driving mode, the steering system is programmed to exert resistive torque feedback like that expected in current autonomous vehicles [48].

The information required by the steering control system to reproduce vehicle driving in autonomous mode in the simulation environment requires the integration and processing of a set of signals such as the torque applied to the steering, the steering angle and centering signal of the steering wheel, the linear displacement of the accelerator and brake pedals, and the force applied to the brake.

The values of these signals are continuously recorded throughout the autonomous driving test route and are logged in a .txt file. This information is stored at a frequency of 10 Hz. The data file contains, in addition to the information from the aforementioned sensors, the coordinates, station, the vehicle’s x, y, and z position, the lateral distance from the road axis, the vehicle’s rotation angle, the vehicle’s longitudinal and lateral speeds, the gear position engaged in the transmission system, the engine revolutions per minute (RPM), the scenario operating readiness zone (manual or autonomous mode), the vehicle’s operating mode (manual or autonomous), and the actions taken by the driver in each driving zone.

With these data, the driver’s behavior can be assessed after any disengagement of the autonomous system. For example, sudden changes in trajectory, harsh braking, or drifting within the lane may be responses derived by unsafe disengagements. A thorough analysis of these events is essential to ensure safe human–machine integration.

As a result, two types of experiments were conducted to calibrate the simulator’s primary controls and to preliminarily assess the EVACH simulator’s autonomous driving system. The first set of tests focused on validating the calibration of the sensors that manage the hand and pedal controls. The second set aimed at examining the extent to which the driving simulator reproduces autonomous driving behavior. Given the limited sample size available for comparison with real-world data, this latter assessment should be regarded as a preliminary analysis. More comprehensive validation of field data will be addressed in future work, also considering how the simulation’s lower variability could be influenced by real-world unpredictability.

**Figure 3:**
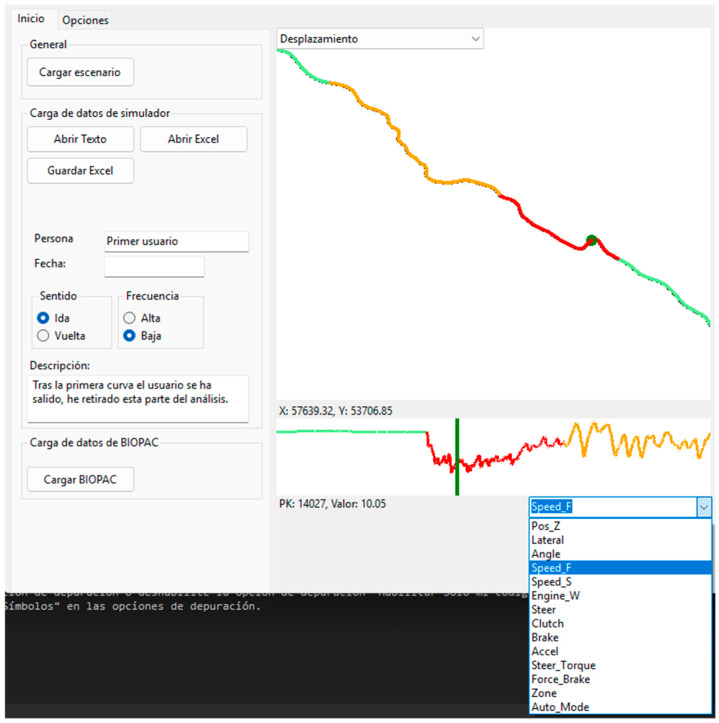
Screenshot of the application showing part of the vehicle trajectory (main window) together with the speed profile. The green zones represent autonomous driving, the red zones correspond to manual driving, and the orange zones represent segments with autonomous capabilities but where the driver chose to manually control the vehicle. The lower profile can display not only the speed, but any of the recorded variables. The app developed for the project displays information about the simulation scenario used in the analysis (**top left corner**), allowing data to be uploaded in text and Excel formats, as well as saved in Excel format (**top left corner**). It also allows data to be analyzed by user (individual simulation), defining the session date, traffic flow, and sampling frequency for the simulation data analysis. It also facilitates the inclusion of comments related to events that occurred during the simulation session (**center left box**). The app also allows data to be uploaded from the BIOPAC biometric data acquisition card, which is analyzed together with the data obtained from the simulation (**bottom left corner**).

A software application was developed under the VB.NET environment, which allowed us to visualize and analyze every single sensor input as a function of the position of the vehicle and driving mode (see Figure 3).

### 2.1. Calibration Methodology of the Primary Controls Used in the EVACH Autonomous Driving Simulator

Two types of tests were developed for the calibration of the simulator primary controls: calibration of the braking system based on the force applied to the brake pedal, and the calibration of the steering control system by measuring the torque applied to the steering wheel.

**Figure 4:**
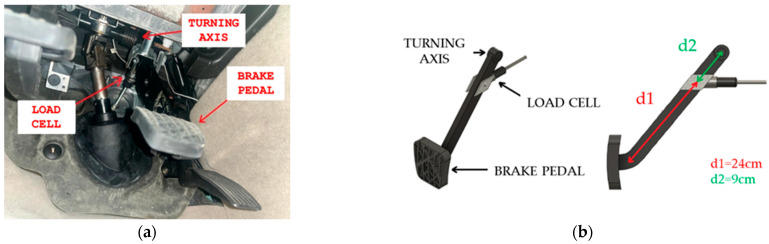
(**a**) Components of the braking system in the EVACH simulator. (**b**) Position of the load cell in the brake pedal of the EVACH driving simulator. Distance d1 (red line) represents the distance from the base of the brake pedal to the load cell position, while distance d2 (green line) represents the distance from the load cell to the brake pedal anchor joint.

For the calibration of the braking system, it was verified that the braking force applied to the brake pedal matched the braking force recorded in the data file obtained in each simulation session. The EVACH simulator is equipped with a load cell sensor that is not located directly above the base of the brake pedal (see Figure 4). Therefore, the actual force applied to the pedal by the driver must be stored by the load cell using a correction factor for the applied effort. In turn, the load cell records the information on the applied force, which is processed, converted, and sent to the data acquisition card, which stores the force value in a .txt data file at a frequency of 10 Hz.

To calibrate the braking system, a Mecmesin [49] force and torque meter was used, capable of accurately measuring up to 500 N of force. Data collection consisted of applying different linear forces to the brake pedal with orders of magnitude close to normal driving to measure significant driving behaviors. Specifically, 10 samples were taken of each of the representative forces: 100, 150, 200, 250, 300, and 350 N.

These representative forces were selected considering that UN/ECE R13-H [50] determines the M1 vehicles’ type-approval requirements regarding the braking systems. This regulation defines the braking equipment to be installed in a motor vehicle as a system that must be designed, manufactured, and installed so that under normal conditions of use, it can stop the vehicle in a controlled, stable, and safe way in the shortest possible distance, regardless of the conditions of vibration to which it may be subjected. The type-approval procedure states that with an M1 vehicle, the mean deceleration must not be lower than 5.8 m/s^2^ and the maximum operative force onto the brake pedal must be equal or lower than 500 N. Nevertheless, all current vehicles have anti-block braking systems (ABS) installed, and therefore, the efforts applied to the pedals are less than the maximum required in the type-approval braking test.

As a result, the pedal forces required for the type-approval procedures do not represent the values usually applied by drivers in current market motor vehicles, as comfort and safety related to the design of the primary controls are considered by the original equipment manufacturers (OEMs) as factors on which commercial confidentiality must be maintained. There have been few research works in the scientific literature presenting results considering the vehicle’s operative forces on primary controls with naturalistic driving, but some data can be obtained from Horberry and Inwood [51] that determined that the average braking efforts on a vehicle with ABS are around 140 N, although some models may need 180–340 N to perform an emergency stop. Some vehicle manufacturers consulted by them determine that, on average, the braking forces needed to stop a vehicle are below a maximum force of 90 N, and in the case of emergency braking, can reach 340–370 N in vehicles without ABS, and lower values for vehicles with ABS (currently all existing).

Also, the authors of [46] developed a research project in which the operational forces applied onto the controls of a single vehicle were measured during different maneuvers representative of normal driving. The study involved 24 subjects distributed by age, gender, and anthropometric measures. Within the testing battery, a sudden braking test of a vehicle equipped with ABS travelling at 50 km/h was performed. The average force on the brake pedal was 240 N, and its minimum value was 111 N.

For this reason, the range of the representative forces selected from 100 to 350 N were considered as real-life braking pedal forces for up-to-date vehicle models.

These forces, recorded by the load cell, were transmitted to the data acquisition card, where they were processed and converted into values that were stored in the data output file. The Results section shows the correlation value obtained. To determine the accuracy of the results, the absolute error of the recorded measurements was calculated between the force applied to the brake pedal and the force recorded in the data file. This error is measured as a percentage of the difference in the data recorded in each case.

**Figure 5:**
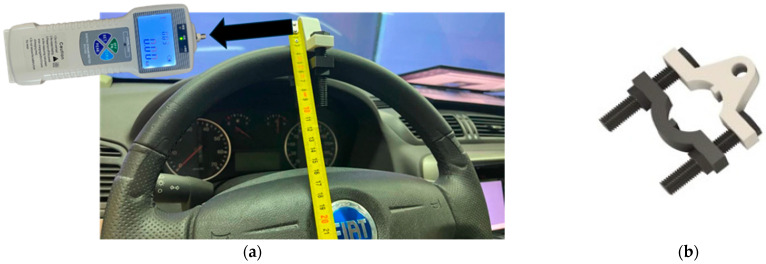
(**a**) Procedure for measuring the tangential force applied to the steering wheel of the simulator with the BAOSHISHAN ZP-500N dynamometer [52]. (**b**) Testing equipment developed for assuring the perpendicularity of the tangential force measurement during the trial testing.

To calibrate the steering system, it was necessary to measure the torque applied to the steering wheel. To this end, an experiment was designed in which different forces were transmitted to the periphery of the steering wheel. To ensure that the tangential force applied to the steering wheel was completely perpendicular to its radius, a special tool was designed using Fusion360 software (Version 16.15.0.2610) and made of ABS plastic that was much more flexible and impact resistant (see Figure 5). This tool can be used to secure the dynamometer position to apply a linear force while ensuring perpendicularity to the steering wheel radius.

This force was converted into torque by the sensor installed in the steering system (see Figure 2, number 5). This value is transmitted to the data acquisition card, whose signal is processed, converted, and stored in the output data file. To record the force applied to the steering wheel periphery, a BAOSHISHAN ZP-500N digital dynamometer [52] was used, capable of transmitting forces of up to 500 N (see Figure 5). Data collection consisted of applying forces in 5 N intervals clockwise and counterclockwise within a range of ±30 N. To determine the accuracy of the results, the absolute error of the recorded measurements between the torque applied to the steering wheel and the torque recorded in the output data file was calculated and compared for each case. The Results section shows the correlation values obtained during the tests.

### 2.2. Pilot Test for the Validation of the EVACH Simulator Steering System Control Software

To validate the steering system control software to ensure that autonomous driving resembled real-life driving conditions, a pilot experiment was designed. The objective was to verify that the developed virtual scenario triggered the same disengagements as a real autonomous vehicle did, at the same location. The rural road on which the virtual scenario is based was modeled by the same authors in previous research using Civil 3D and a multilayer editing procedure (MEP). This editing methodology consists of a multilayer addition technique in which different data files incorporate the types of information necessary to generate the road and its surrounding virtual environment, which was validated in [53,54].

This MEP methodology allows virtual reality urban and interurban environments of more than 100 km^2^ to be built, instead of other 3D modeling market tools such as 3DSmax (version 2016) or Blender (version 4.08.01.0810), which suffer from technical limitations such as an excessive amount of memory required or limited precision floating-point numbers. The programming environment developed in this scenario was based on Visual C++ (version 4.08.01.0810) Express to run in real-time. To generate meshes of objects in the environment, such as signs, walls, trees, etc., the 3D modeling Blender 2.70 was used. Python 3 was used to process the data and calculations offline.

The analyzed road segment corresponds to the CV-35, from Losa del Obispo to Titaguas, located in the Valencian Community (Spain). This is a regional interurban road with a total length of 29.3 km, a cross-section with an average lane width of 3.25 m, and a 0.25 m shoulder. This road was selected because several zones with different geometric characteristics could be identified, covering a wide range of tangents, curves, longitudinal grades and representing the different operating states of the high-speed vehicles.

The cartographic files were obtained based on a LiDAR with a density of 5 m mesh size. The horizontal alignment was obtained according to the methodology proposed by [55]. In addition, the vertical alignment was recreated using Civil 3D based on altitude information provided by the global positioning system (GPS) data of the tests. The orthophotography was downloaded free of charge from the National Aerial Orthophotography Plan (PNOA) website. An inventory of road and surrounding elements was also compiled and completed in an Excel file containing all superstructure elements such as vertical signs, road markers, road markings, and side safety barriers.

To support vehicle dynamics and their behavior with the collision system, we used NVidia PhysX library 3.3. This is an open-source physics solution that takes advantage of handling large virtual environments, as was our case. The audiovisual section rests with the Microsoft DirectX 8.1 libraries specifically for Direct3D graphics rendering and DirectSound (version 4.08.01.0810) for sound reproduction. The Nvidia PhysX libraries operate at 60 Hz. This frequency is considered sufficiently accurate to process the data information transmitted by the simulator’s data acquisition card.

Due to the technical characteristics of the autonomous driving considered in the pilot tests, the vehicle dynamics model applied consisted of a point-mass model [56], in which the vehicle is treated as a mass concentrated at its center of gravity (CG), with all the forces acting on the CG as primary factors that deliver the vehicle’s motion. The two degrees of freedom (DOF) model includes exclusively the longitudinal and lateral movement of the CG, excluding the lateral forces appearing during changes in vehicle orientation and not considering the interaction between the vehicle and the road throughout tire slip and grip.

This point-mass model represents the simplest vehicle model allowing scenarios with the aim of speed optimization, where the controller can easily track the longitudinal and lateral accelerations of the vehicle. This model has the advantage of demanding the least computational resources, making it an optimal solution for real-time simulations.

The technical characteristics of the vehicle used for the virtual scenario experiment was based on a Ford Escort XR3 vehicle (FORD SPAIN, Almussafes, Valencia, Spain) with a curb weight of 1380 kg, 4 × 2 front-wheel drive, a length of 4.06 m, and a center of gravity height of 0.503 m. It was equipped with 185/60 R14 tires, McPherson strut front and rear suspension, a 1998 cc engine with a maximum power of 120 hp and a maximum torque of 289 Nm, a 5-speed automatic transmission (Rt1 = 2.981; Rt2 = 2.07; Rt3 = 1.539; Rt4 = 1.198; Rt5 = 0.975), and a final drive ratio of 4.059.

The specific driving modes within each zone were established based on the results previously obtained from a naturalistic test conducted on the study road (CV-35) with a SAE 2 level vehicle. The vehicle selected to carry out the naturalistic tests was a 2018 Audi Q2. This vehicle has technical characteristics very similar to those of the Ford Escort XR3 used in the previously validated simulator. It has a curb weight of 1330 kg, 4 × 2 front-wheel drive, a length of 4.2 m, a gasoline engine with a maximum power of 116 hp at 5500 rpm, a maximum torque of 200 Nm, and a 6-speed manual gearbox.

The point-mass vehicle dynamic model used in the driving simulation has been validated in previous research [53,54]. Although the Ford Escort XR3 is a vehicle model older than the 2018 Audi Q2 and is not equipped with ADAS driver assistance systems that facilitate autonomous behavior such as SAE2, the advantage of the Audi Q2 compared with the simulated model is that it is equipped with the ACC system and a lane assistive system (LAS) that helps the driver keep the vehicle in the lane with gentle steering interventions. This system is activated at speeds of 65 km/h (40 mph).

However, since these vehicles have very similar construction characteristics—curb weight, dimensions and transmission—the results obtained when considering an autonomous driving mode and a simple point-mass vehicle dynamics model do not differ much in terms of the speed profile and the trajectory followed on the route.

Nevertheless, this aspect constitutes a limitation of the study carried out that should be improved in successive extensions of the work to include driving with other SAE 2 vehicles and to incorporate SAE 3 vehicles in the validation of the simulator’s dynamic model.

To develop the naturalistic driving pilot test, the vehicle was equipped with three GPS-equipped cameras to continuously record the altitude, latitude, longitude, and speed. The system also identified the status of the autonomous driving system, locating the points where the system disconnects and reconnects.

The information recorded in the field was processed and stored in a database that includes, in addition to the variables of the road geometry, cross-section, and road markings, the level of engagement of the SAE2 level system during the tests (connected/disconnected).

Two analyses were carried out to check the ability of the simulator to reproduce the manual/autonomous performance. The first analysis compared the average speed and its dispersion along several autonomous zones. The second one focused on variations in speed during transitions from autonomous to manual mode.

The real-world road segment under study presented many locations where the vehicle disengaged, leading to up to 29 autonomous zones. Not all of these zones were adequate for the analysis; these had to be refined following these criteria:

- Sections should be long enough to prevent biasing from the preceding speed;
- Sections should present variability in horizontal alignment;
- Sections should present variability in vertical alignment.

Taking these criteria into account, up to seven different sections were selected with the following characteristics (all stations refer to 0 as the beginning of the global road segment):

- Section 1: From station 2 + 270 to 4 + 730, characterized by long tangents and a downgrade;
- Section 2: From station 8 + 350 to 12 + 030 m with large horizontal curves and upgrades;
- Section 3: Located immediately after Section 2, ranging from station 12 + 150 to 13 + 610 consisting of a tangent in an upgrade alignment;
- Section 4: From station 18 + 140 to 19 + 510 featuring a long tangent and diverse vertical alignment;
- Section 5: From station 21 + 850 to 22 + 910 with a tangent in an upgrade section;
- Section 6: From station 24 + 190 to 25 + 220 with characteristics like Section 5;
- Section 7: From station 28 + 710 to 29 + 290 comprising a horizontal curve and a downgrade vertical alignment.

**Figure 6:**
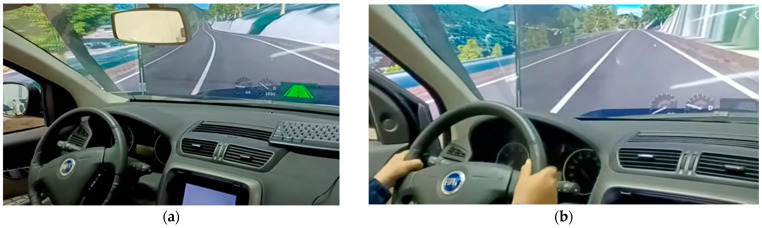
(**a**) Green icon on screen indicates autonomous mode. (**b**) Gray icon represents manual mode.

Five volunteers participated in the study, driving the simulator along the scenario. Similarly to actual vehicles, the simulator indicated whether it was running in automated mode or not, using a green, road-shaped icon (Figure 6a). This icon turned gray when the autonomous system was disengaged (i.e., manual mode) (Figure 6b).

All volunteers participating in the experimental validation tests signed the corresponding consent and authorization for the use of their personal data, granted by the Ethics Committee of Universitat Politècnica de València (Reference P02_27-11-2024).

The simulation experiments began with a warm-up section where the volunteers drove in manual and autonomous mode. To engage the vehicle’s autonomous mode, the driver had to drive through an autonomous-ready zone and increase the speed above 60 km/h for about two seconds. From this point on, the steering wheel performed automatically, with the driver only having to keep their hands on the wheel with no manual torque (SAE level 2).

Disengagement zones can be set up by researchers in advance. These were chosen to be similar to reality. When reaching one of these zones, the simulator disengages: the simulator (vehicle) emits a sound, the steering wheel stops turning autonomously, and speed begins to decelerate (as if the accelerator pedal had been released). Volunteers must then take over by applying torque to the steering wheel and operating the pedals to keep the vehicle in the lane. Along with autonomous zones, volunteers could also take control by pressing the brake pedal or controlling the steering wheel.

This takeover behavior of the manual/autonomous driving zones is reproduced sequentially throughout the entire virtual scenario, following the same sequence obtained during naturalistic driving for all sections defined on the CV-35 road mapped in the study.

The data recorded in these experiments were compared with the information from real-world data for all seven sections. The first 100 m of each section was removed from the final data to avoid the influence of the speed prior to activating autonomous mode. Therefore, for each of the selected autonomous driving sections, the average speeds of each simulation session were compared with the average speeds of the same section in naturalistic driving. This analysis was completed with the statistical value of the standard deviation and the coefficient of variation of the recorded signals.

## 3. Results and Discussion

### 3.1. Calibration of the EVACH Driving Simulator Braking System

To perform the braking system calibration tests, various forces of magnitude representative of normal vehicle driving were applied to the brake pedal [50,51], values corresponding to 100, 150, 200, 250, 300, and 350 N. Ten samples were taken for each applied force, and the data were recorded both on the brake pedal and in the output file of the virtual simulation program. The results of the tests are shown in Table 1. This table shows the target forces to be measured in each of the tests. Each row of the table presents, for each of the samples taken, the value of the forces measured directly on the brake pedal with the MECMESIN digital dynamometer [49] and the force recorded in the results output file once the signal has been processed and converted by the data acquisition card. Finally, the error, in absolute terms, incurred during the measurement of the two signals is expressed as a percentage value.

Considering the calibration of the braking system, as shown in Table 1, the brake calibration errors were generally within 2.7%. It was detected that higher errors appeared at the lower forces applied onto the braking pedal. This error could be attributed, on the one hand, to the differences in the sampling frequency of the force dynamometer, which was 5000 Hz [37], compared with the data recording frequency in the output file, which was only 10 Hz. This error could also be due to the sample forces applied to the pedal base at a different orientation, not exactly perpendicular to the pedal central bar, which could have influenced the measurement taken in the load cell sensor (see Figure 3), where the signal is sent to the data acquisition card, post-processed, and recorded in the data file during the simulation.

As the comparison of the results obtained was purely descriptive, the magnitude of the error detected was small enough to consider that the actual effort applied to the simulator braking pedal was close enough to the measurement recorded in the results file after all the internal processing at different frequencies in different stages of data processing.

### 3.2. Calibration of the EVACH Driving Simulator Steering System

The experiment developed to calibrate the torque applied to the steering system consisted of applying a force to the periphery of the steering wheel. This force, after being converted into torque by multiplying it by the distance from the center of the steering wheel (21 cm), was recorded by the torque sensor and processed on the data acquisition card. This torque was then compared with the torque value recorded by the steering control system in the data output file.

The forces applied to the periphery of the steering wheel (see Figure 5a) varied within a range of ±30 N, in 5 N intervals, and were applied individually in clockwise and counterclockwise directions. The results of the measurements obtained by the torque sensor installed in the steering system and the torque recorded by the control system are shown in Table 2. Table 2 presents in each row, and for each of the samples taken, the steering wheel target force to be measured in the periphery of the steering wheel, the value of the steering wheel peripheral forces applied by the BAOSHISHAN ZP-500N dynamometer [52], expressed in Newtons (N) and converted to kilogram-force (kgf), the torque sensor measurement obtained in the torque load cell (see Figure 2, number 5) expressed in Nm, and the torque data registered in the output file once the signal has been processed and converted by the data acquisition card (expressed in Nm). The last column shows the error, in absolute terms, incurred during the measurement of the two torque signals expressed as a percentage value.

In relation to the steering wheel control system, the results obtained in the calibration tests showed that the order of magnitude of the maximum torque recorded by the control system during the test trials was approximately ±6.3 Nm, a value very close to that obtained in current market standard cars [46,47,48]. The maximum error detected between the torque measurements made by the torque sensor in the steering system and the torque recorded by the control system, once the information was processed on the data acquisition card, did not exceed 5.95%. It was found that the greatest errors appeared with lower force applied to the steering wheel, generating a difference between the measured torques of 0.1 Nm, which is considered a negligible order of magnitude in relation to the driving conditions of vehicles on the current market [47,48].

The deviation in the data recorded may be due to the different sampling frequencies of the measurement systems: approximately 1000 Hz for the force dynamometer used to apply forces to the steering wheel [52] and 10 Hz for recording information in the results file. This error is practically negligible during driving tests in autonomous mode and can only influence manual driving mode, so it can be concluded that the steering control system of the EVACH driving simulator is perfectly calibrated to reproduce different driving modes of autonomous vehicles in TOR operations.

### 3.3. Preliminary Analysis of the Virtual Driving Scenario in Autonomous Mode of the EVACH Driving Simulator

The results of the comparative analysis between the average speeds on each section selected for autonomous driving in the virtual scenario of the EVACH simulator and the corresponding speed recorded on a similar section of the CV-35 during the naturalistic driving test can be seen in Table 3. This table shows the average vehicle speed while traveling on each section in real driving and autonomous mode, the standard deviation, and the coefficient of variation. The average speed, standard deviation, and coefficient of variation were obtained with Equation (1), Equation (2), and Equation (3), respectively:

(1) $$\bar{v}=\frac{\sum_{i=1}^{N}v_{i}}{N}$$

(2) $$s=\sqrt{\sum \frac{{\left(v_{i}-\bar{v}\right)}^{2}}{N-1}}$$

(3) $$CV=\frac{s}{\bar{v}}\;\;\;if\;\bar{v}\neq 0$$

where $\bar{v}$ is the average speed, $v_{i}$ represents each of the data for speeds recorded along each section tested, N is the total number of speed data obtained in each section analyzed, s is the standard deviation, and CV is the coefficient of variation.

**Figure 7:**
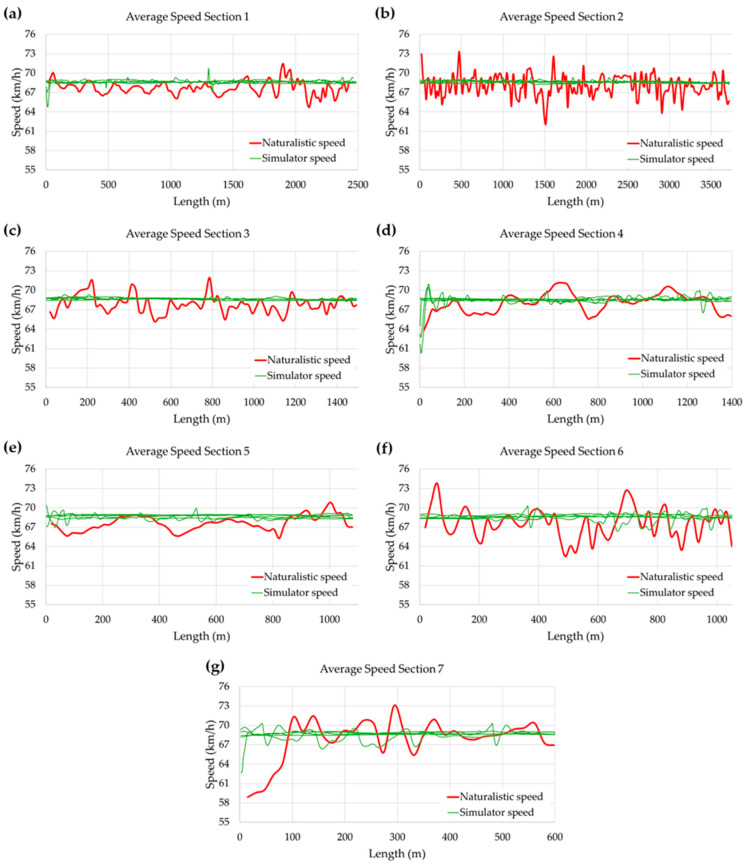
(**a**) Comparison between the average speed recorded in Section 1 of the CV-35 (red line) and the average speed recorded in each one of the virtual simulation sessions (green lines) recorded in the simulation data output file in Section 1; (**b**) Idem. Section 2; (**c**) Idem. Section 3; (**d**) Idem. Section 4; (**e**) Idem. Section 5; (**f**) Idem. Section 6; (**g**) Idem. Section 7.

Figure 7 shows the average speed logs of the seven sections analyzed on the CV-35 road obtained during the naturalistic tests (red line) for each section, ranging between 65 and 71 km/h. The sampling rate for the autonomous vehicle’s speeds during naturalistic driving was 1 sample per second. The average speeds recorded on each section of the real-life circuit were obtained by analyzing the speed logs of an SAE 2 autonomous vehicle traveling on the global CV-35 road segment.

The average speed obtained in all sections of the virtual environment for each of the five participants in the simulator test can be seen in green in Figure 7. The frequency of data recording was 10 Hz, and the framerate for the time-step calculus in the simulator vehicle dynamic model was 60 Hz, which is considered accurate enough for processing all of the data obtained during the session tests.

As the logic core of the system was based on an STM32 board, where the control algorithm programmed in C language compares the recorded position and torque in real-time with the target values from the simulator scenario, the steering system control dynamically adjusts, in real-time, the signal sent to the motor assuring the correct behavior of the autonomous driving mode at the simulator.

**Figure 8:**
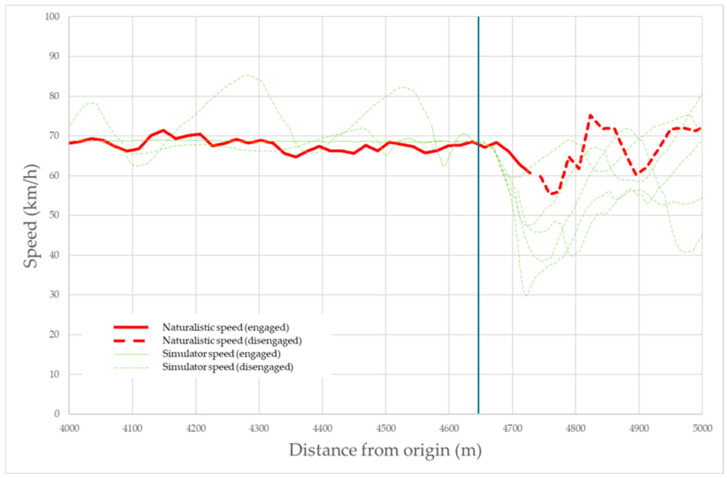
Comparison of all five volunteer (green) and the real-world (red) speed profile patterns when a disengagement takes place. Solid lines mean that the volunteer was driving in autonomous mode (dashed lines: manual mode).

As mentioned before, the shift from autonomous to manual driving is of major interest given its relationship to road safety. Thus, the driving simulator was proven to show how the speed varied when disengagements took place (Figure 8). All volunteer speed profiles presented sudden deceleration and a higher speed variation when taking over. The same applies to real-world vehicles, with a slightly lower speed of variation.

**Figure 9:**
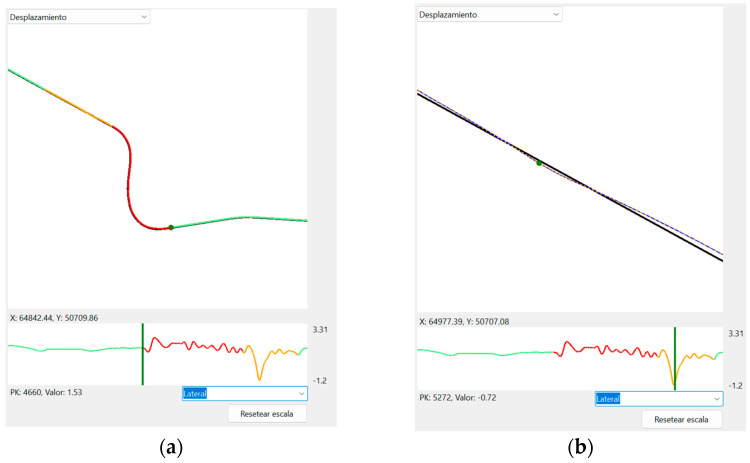
The EVACH project software application is also able to analyze the lateral distance from the road axis. (**a**) Lateral distance profile in which a disengagement causes the driver to take control of the steering wheel (red zone), presenting a quite different pattern than in autonomous mode (green). (**b**) Detail of how the driver trajectory (orange dots) and the road axis (black line) are shown when zooming in.

In addition to speed variation, disengagements also cause sudden variations in the vehicle’s lateral position. A higher-frequency lateral position correction can be seen when running in manual mode (Figure 9). A comparison between these lateral position patterns between the real-world and simulation test would be of high interest. However, it requires more accurate real-world data and will be carried out in further research.

In the pilot testing validation process of the virtual simulation scenario for autonomous driving, it was found that the average speeds obtained in the naturalistic tests across the seven sections analyzed ranged from a minimum of 67.3 km/h (Section 6) to a maximum of 68.7 km/h (Section 7). In other words, during autonomous driving on a real road, vehicle speeds differ by a maximum of 1.4 km/h. This means that during naturalistic driving, the autonomous driving system behaved in a very similar way, regardless of the varying geometric conditions of the terrain, managing to stabilize the average speed of the vehicle within the limits established for the operation of the autonomous driving system.

The dispersion of the data obtained for the average speed in each road section was also reduced, obtaining a maximum standard deviation of the average speeds of 2.2 km/h in Section 6, equivalent to a coefficient of variation of 3.3%.

Fluctuations in realistic average speed may be due to the appearance of endogenous factors of the vehicle (type of transmission, condition of the tires, condition of the body, resistance to movement, etc.) and other exogenous factors that influenced the variation in average speed in each section such as the appearance of irregularities in the road, different coefficients of friction on the asphalt (humidity, gravel, water, etc.), crosswind that increases resistance to movement, or even errors in GPS measurements.

On the other hand, analyzing the results observed in Figure 7 for all sections of the virtual environment, the average speed in autonomous mode varied between a minimum value of 68.55 km/h to a maximum of 68.67 km/h (records in green in Figure 7a–g plots), with maximum standard deviations of 0.48 km/h and a maximum coefficient of variation of 0.7% (Section 7). In each of the average speed records obtained for each of the volunteer subjects who participated in the tests and for each of the autonomous driving sections, a very similar behavior was observed, where the average speed recorded remained at values slightly lower than 69 km/h throughout the entire driving in the virtual scenario.

This made the average speed recorded in autonomous simulation mode in the EVACH simulator much more uniform and with fewer fluctuations throughout the entire section. This could be because driving in the virtual simulator environment allows for optimal road conditions (optimal grip, absence of crosswinds, minimal rolling resistance, etc.), which can be defined a priori in the virtual environment design model and do not undergo changes due to variable external conditions along the route (wind, rain, traffic, etc.).

In general terms, when comparing the average driving speeds profiles in autonomous mode between on-road driving and the virtual scenario, for each of the analyzed sections, it was observed that the speed in the virtual scenario was typically at most 1 km/h higher than that obtained during real-life driving on the equivalent section. A comparative analysis of the speeds obtained shows that the data dispersion in the virtual simulation was much lower than that obtained in naturalistic driving, with a coefficient of variation of 0.7% maximum in the autonomous driving simulation compared with 3.2% maximum in naturalistic driving. This aspect underscores the stability and homogeneity of autonomous driving in the simulator’s virtual scenario.

The simplicity of the single-mass approach used in the dynamic vehicle model could contribute to reduce the induced errors due to exogenous conditions of the simulations (absence of lateral forces during changes in vehicle orientation and no consideration of tire slip and grip). This would need to be improved in further research and constitutes an actual limitation in the accuracy of the driving behavior of the steering simulation control in autonomous mode.

Nevertheless, compared with other similar autonomous driving studies [35,37,38,39] where an autonomous driving simulator was used as an experimental tool in the research, with closed-design hardware and software that equipped predefined haptic or modeling characteristics that could not be modified, or have not been experimentally validated, the EVACH simulator could be used as a partially validated experimental tool for conducting controlled autonomous driving experiments in virtual reality interurban scenarios of more than 100 km^2^ specifically designed for evaluating driver behavior in variable traffic environments.

## 4. Conclusions

Hardware and software customization was a key strategy for the development of this research project, allowing for optimized performance of the steering control system and ensuring efficient integration of all subsystems. The implementation of a custom-made data acquisition system has made it possible to precisely adapt the sensors and actuators to the needs of the EVACH autonomous driving simulator, optimizing the detection and acquisition of critical data for analyzing vehicle behavior in autonomous mode.

Overall, the results obtained from the calibration of the EVACH simulator sensors demonstrate that the combination of custom-designed hardware, an efficient acquisition system, and the use of advanced validation tools has resulted in a robust and scalable platform.

The results presented in this article show that the calibration of the simulator’s primary controls met the targeted accuracy, allowing for the faithful reproduction of maneuvers performed during both manual driving, and above all, in autonomous mode. One of the advantages of the MEP methodology applied for designing virtual scenarios in the EVACH simulator is that can be used for conducting controlled autonomous driving experiments in virtual reality interurban scenarios of more than 100 km^2^ specifically designed for evaluating driver behavior in variable traffic environments.

As a result, the experimental validation developed for the virtual scenario allows this simulator to be used as a versatile tool in the research and analysis of autonomous driving at the SAE 2 and SAE 3 automation levels as well as for the development of future similar projects.

However, some limitations must be acknowledged. As a fixed-base simulator, the EVACH simulator does not provide motion feedback, which may influence certain perceptual aspects of the driving task compared with real-world conditions. In addition, although its scenarios are based on naturalistic data, they cannot fully reproduce the complexity and variability of real traffic environments. Potential sources of error, such as the calibration of sensors and the simplification of external variables, should also be considered.

Another limitation of the research undertaken consists of the simplicity of the single-mass approach used in the dynamic vehicle model, which contributes to a minor accuracy in the results due to the absence of lateral forces acting during changes in vehicle orientation and no consideration of tire slip and grip. This aspect will be improved in further research and constitutes an actual limitation in the analysis of driving behavior in autonomous mode.

Nevertheless, these limitations do not undermine the contributions of the simulator but rather define the context in which its results should be interpreted. Future research will address these aspects by extending the range of scenarios and incorporating additional validation studies to further enhance the ecological validity of the experimental platform.

## Figures and Tables

**Table 1:** Values of the forces applied to the brake pedal relative to the forces recorded in the data output log.

**Brake Force Target (N)**	**Brake Pedal Force Applied (N)**	**Output File Force Registered (N)**	**Error (%)**	**Brake Force Target (N)**	**Brake Pedal Force Applied (N)**	**Output File Force Registered (N)**	**Error (%)**
100	92.82	92.50	0.32	150	140.08	138.02	2.06
	93.51	95.01	1.50		144.12	145.51	1.39
	94.68	93.52	1.16		146.11	145.02	1.09
	96.88	98.02	1.14		148.65	149.53	0.88
	98.08	99.51	1.43		151.55	153.01	1.46
	100.13	98.03	2.10		153.77	155.54	1.77
	102.77	104.52	1.75		156.29	158.03	1.74
	103.22	100.51	2.71		158.02	156.51	1.51
	105.15	106.01	0.86		159.53	156.97	2.56
	106.75	105.53	1.22		160.59	161.46	0.87
Mean value	99.40	99.32	1.42	Mean value	151.87	151.96	1.53
**Brake Force Target (N)**	**Brake Pedal Force Applied (N)**	**Output File Force Registered (N)**	**Error (%)**	**Brake Force Target (N)**	**Brake Pedal Force Applied (N)**	**Output File Force Registered (N)**	**Error (%)**
200	190.40	193.50	1.63	250	238.64	237.02	1.62
	193.10	195.00	0.98		244.70	245.40	0.70
	197.00	194.50	1.27		247.61	248.50	0.89
	198.20	201.00	1.41		248.63	249.03	0.40
	200.70	202.00	0.65		250.70	246.51	1.68
	202.60	200.50	1.04		252.66	253.02	0.36
	205.10	202.50	1.27		257.80	259.01	1.21
	208.40	210.00	0.77		260.09	261.53	1.44
	210.90	213.00	1.00		257.10	258.02	0.92
	212.20	214.50	1.08		262.58	263.50	0.92
Mean value	201.86	202.65	1.11	Mean value	251.71	252.15	1.01
**Brake Force Target (N)**	**Brake Pedal Force Applied (N)**	**Output File Force Registered (N)**	**Error (%)**	**Brake Force Target (N)**	**Brake Pedal Force Applied (N)**	**Output File Force Registered (N)**	**Error (%)**
300	290.75	293.02	0.76	350	344.96	343.51	1.45
	293.12	295.48	0.82		340.49	339.50	0.99
	296.81	292.03	1.62		345.18	348.02	0.81
	298.48	294.97	1.17		348.08	350.48	0.69
	300.09	302.52	0.80		350.21	348.49	0.49
	302.91	305.51	0.86		352.92	355.01	0.60
	305.42	301.01	1.44		355.02	362.03	1.97
	308.21	310.45	0.75		358.28	356.48	0.50
	310.60	312.01	0.45		362.85	364.98	0.58
	312.49	307.00	1.76		365.56	366.50	0.25
Mean value	301.89	301.40	1.04	Mean value	351.97	353.50	0.83

**Table 2:** Comparison between the torque applied to the simulator steering wheel and the steering system torque recorded in the output data file.

Steering Wheel Force Target (N)	Steering Wheel Force Applied (N)	Steering Wheel Force Applied (kgf)	Torque Sensor Measurement (Nm)	Torque Output File Registered (Nm)	Error (%)
−30.0	−30.2	−3.079	−6.342	−6.288	0.85
−25.0	−24.5	−2.498	−5.145	−5.021	2.41
−20.0	−18.8	−1.917	−3.948	−3.859	2.25
−15.0	−15.2	−1.550	−3.192	−3.236	1.37
−10.0	−11.0	−1.122	−2.31	−2.279	1.34
−5.0	−5.3	−0.534	−1.113	−1.137	2.15
5.0	5.2	0.530	1.092	1.027	5.95
10.0	9.5	0.969	1.995	1.897	4.91
15.0	14.5	1.479	3.045	2.931	3.74
20.0	19.2	1.958	4.032	4.101	1.71
25.0	24.8	2.529	5.208	5.162	0.88
30.0	29.7	3.028	6.237	6.306	1.10

**Table 3:** Comparison of the average speeds ($\bar{v}$) obtained for each section of autonomous driving in the virtual scenario of the EVACH simulator with those obtained for each section of the CV-35 road in naturalistic driving. s is the standard deviation, and CV is the coefficient of variation.

	CV-35 Real Road Data			Virtual Scenario Data		
	$\bar{v}$ (km/h)	s (km/h)	CV (%)	$\bar{v}$ (km/h)	s (km/h)	CV (%)
Section 1	67.662	1.009	1.492	68.634	0.184	0.268
Section 2	67.959	1.623	2.388	68.618	0.170	0.247
Section 3	67.825	1.358	2.002	68.633	0.154	0.224
Section 4	68.155	1.431	2.100	68.558	0.265	0.387
Section 5	67.627	1.144	1.691	68.673	0.268	0.391
Section 6	67.346	2.221	3.298	68.613	0.426	0.622
Section 7	68.763	1.911	2.779	68.671	0.485	0.706

## Data Availability

The authors do not have permission to share data.

## Abbreviations

The following abbreviations are used in this manuscript:

ABS	Anti-block braking systems
ACC	Adaptive cruise control
AV	Autonomous vehicles
CDC	Communications device class
CG	Center of gravity
DOF	Degrees of freedom
GPS	Global positioning system
LAS	Lane assistive system
LCA	Lane centering assist
LKA	Lane keeping assist
MEP	Multilayer editing procedure
NDRT	Non-driving-related tasks
ODD	Operational design domain
OEM	Original equipment manufacturer
PK	Kilometer point
PNOA	National Aerial Orthophotography Plan
RPM	Revolutions per minute
RSA	Research Spanish Agency
SAE	Society of Automotive Engineers
TOR	Take-over request
USART	Universal synchronous/asynchronous receiver/transmitter
